# Potential Impact of SARS-CoV-2 Spike Protein on HIV-1 Reservoir in People Living with HIV

**DOI:** 10.3390/v18020154

**Published:** 2026-01-23

**Authors:** Maurizio Federico

**Affiliations:** National Center for Global Health, Istituto Superiore di Sanità, 00161 Rome, Italy; maurizio.federico@iss.it

**Keywords:** HIV-1 reservoir, mRNA COVID-19 vaccines, SARS-CoV-2 Spike, people living with HIV

## Abstract

People living with HIV-1 (PLWH) are part of the so-called “fragile” populations to which COVID-19 vaccines were/are strongly recommended. The fact that most widely used COVID-19 vaccines rely on the production of a biologically active SARS-CoV-2 Spike protein expressed by synthetic mRNA poses the relevant question of whether and how this vaccination influences the fate of the HIV-1 reservoir. This report presents a detailed analysis of the literature data on the effects of SARS-CoV-2 Spike and COVID-19 vaccines on HIV-1 latently infected cells. Despite being limited in number, the experimental evidences consistently indicate that vaccine mRNA and/or SARS-CoV-2 Spike can effectively reactivate latent HIV-1. This conclusion has been drawn after “in vitro”, “ex vivo”, and “in vivo” assays, and with virus-associated Spike, soluble Spike, or its intracellular expression, as well as with COVID-19 mRNA vaccines. On the other hand, real-world observations on vaccinated PLWH under antiretroviral therapy (ART) provided evidence of HIV-1 reactivation almost exclusively in PLWH with unsuppressed viremia, as measured in terms of size of the HIV-1 reservoir. Although several issues still need to be clarified through urgent additional investigations, these data suggest the possibility that the Spike protein and/or the vaccine mRNA molecules affect the HIV-1 latency in PLWH.

## 1. Introduction

The injection of current mRNA-based COVID-19 vaccines leads to the production of a biologically active, full-length SARS-CoV-2 Spike protein stabilized through two proline mutations at amino acid positions 986 and 987.

The results from a substantial body of literature support the idea that the SARS-CoV-2 Spike protein exhibits strong biological activity in humans regardless of its biological presentation, i.e., whether associated with the viral particle, in its soluble form, or embedded in the cell membrane [1]. For instance, the SARS-CoV-2 Spike protein can generate immunologic imbalances, leading to the induction of autoantibodies, anti-idiotype antibodies, and dysregulated production of various cytokines [2].

On this basis, people with already diagnosed immunologic pathologies should carefully evaluate the possible consequences of the expression of SARS-CoV-2 Spike following vaccination. People living with HIV-1 (PLWH) certainly belong to the category of immunocompromised subjects, even if current antiretroviral therapies (ARTs) allow the reconstitution of an immune system that is generally effective enough to counteract, in the absence of additional pathologies, the consequences of microbial infections. Nevertheless, PLWH have priority over healthy people in terms of access to COVID-19 vaccination, being part of the so-called “fragile” populations.

In PLWH, a small percentage of cells harbor silent forms of HIV-1 integrated into their DNA, albeit only a minor part of them codes for a potentially infecting HIV-1. The antiviral effects of ART guarantee the containment of residual, still active HIV-1 genomes. Occasionally, some factors, including temporary failure of the immune system, can generate the so-called viral “blips”, i.e., low-level, reversible increases in plasma HIV-1 RNA [3,4].

In this scenario, the immunologic equilibrium in PLWH can be best preserved by avoiding the presence of any factor that would favor the reactivation of HIV-1 from its latent form. On this subject, several scientists have asked whether the SARS-CoV-2 Spike protein and/or the COVID-19 mRNA vaccines could influence HIV-1 latency. A comprehensive picture of current scientific evidence was obtained by interrogating the PubMed database regarding the effects of SARS-CoV-2 on the HIV-1 reservoir. Here, a comparative analysis of the results from these studies is presented. Experimental evidence from both “in vitro” and “ex vivo” analyses supports the hypothesis that both intra- and extracellular Spike can switch mechanisms, ultimately leading to HIV-1 reactivation. A critical evaluation of clinical observations carried out in COVID-19 vaccinated PLWH, which yielded apparently inconsistent results, is also provided.

## 2. The HIV-1 Reservoir

ART has saved the lives of millions of people infected with HIV-1. Currently, it consists of a personalized mix of drugs, including both nucleoside and non-nucleoside inhibitors of the HIV-1 reverse transcriptase enzyme, inhibitors of virus entry, integrase strand-transfer inhibitors, and inhibitors of the viral protease [5]. Unfortunately, despite its high potency in blocking the HIV-1 replication, the virus can persist for decades in the form of the so-called viral “reservoir”. It is composed of cells that are latently infected by HIV-1 and harbor the viral genome in their DNA, including essentially all functional and phenotypic classes of CD4+ T lymphocytes [6], tissue-resident macrophages, and brain microglia [7]. A complex of causes contributes to the reduced/undetectable virus production from the HIV-1 reservoir. It comprises the integration of defective and/or hypermutated proviral copies, the integration of the provirus in heterochromatin DNA regions, and cell-mediated mechanisms that negatively control proviral expression. It was estimated that PLWH under ART harbor a provirus in approximately 1 out of 2000 HIV-1 susceptible cells. Among these, only about one out of ten cells integrate a replication-competent HIV-1 genome, ready to spread if ART is discontinued [8].

Recent autopsy studies have shown that the HIV-1 reservoir expands to essentially all tissues and organs, as proven by the finding of intact HIV-1 *env* DNA sequences in as many as 28 anatomic sites [9]. To evaluate the genetic integrity of the persistent proviruses, Dufour and coll. analyzed both HIV-1 DNA and RNA, and carried out nearly full-length (NFL) proviral sequencing in samples from 15 tissues of two ART-treated PLWH who donated their bodies “post-mortem” [10]. HIV-1 DNA sequences were detected in all tissues considered, with the lowest levels measured in the brain and testes, and the highest frequencies of HIV-1 sequences found in secondary lymphoid organs. Intact DNA proviral sequences were found in spleen and mediastinal lymph nodes.

Tang and coll. found full-length, integrated HIV-1 DNA as well as cell-associated HIV-1 RNA in viable microglia isolated from brain tissue after rapid autopsies [11]. Another study demonstrated that lymph node dendritic cells harbor HIV-1 integrated DNA as well as inducible replication-competent virus despite years of suppressive ART [12]. This evidence supports the hypothesis that low-level and/or cell-to-cell infection can occur continuously in ART-suppressed PLWH, most likely in compartments where the efficacy of the antiviral therapy is suboptimal, such as lymphoid germinal centers and the central nervous system.

In sum, ART allows PLWH to experience a near-healthy life, despite a significant number of cells diffused in nearly all districts harboring proviral forms of HIV-1. Part of these HIV-1 genomes are ready to spread by discontinuing ART and/or under stimulatory conditions, with obvious pathogenic effects.

## 3. Vaccine Spike: Biodistribution and Pharmacokinetics

The original idea that the COVID-19 vaccine does not diffuse from the site of inoculation, thereby undergoing rapid degradation, has been largely overtaken. A former outstanding study carried out in humans showed the persistence of both the mRNA and Spike protein in ipsilateral axillary core lymph node biopsies through results obtained by in situ hybridization and immunohistochemical analyses [13]. Concerning the vaccine mRNA, more than 50,000 probe spots/mm^2^ of lymph node tissue were found 37 days after the second dose of the BNT162b2 vaccine, and about 1000 probe spots/mm^2^ lasted at day 60 after vaccination. Meanwhile, the authors highlighted the presence of relevant amounts of cell-free Spike in the same tissue specimens. Finally, a concentration of Spike in plasma of up to 174 pg/mL two days after injection was reported.

A more recent study extends the presence of circulatory Spike from 473 to 709 days after the last vaccination in 4 out of 16 subjects not experiencing SARS-CoV-2 infection [14].

Even more strikingly, the presence of Spike-expressing CD16+/CD14− (non-classical) monocytes was recently reported in 11 out of 12 subjects suffering from post-COVID-19 vaccine syndrome (PCVS), i.e., a condition characterized by long-term symptoms similar to the long-term course of COVID-19, and affecting a small percentage of vaccine recipients [15]. None of the 11 vaccinees seemed to be infected with SARS-CoV-2. The presence of Spike in non-classical monocytes was found by flow cytometry and confirmed by liquid chromatography/mass spectroscopy analysis after cell sorting. Spike-positive cells persisted for up to 245 days after the last injection [16].

Taken together, these data support the idea that vaccine Spike can persist for variable, yet extended periods of time. Even if the underlying mechanism is still unknown, most likely, this persistence is associated with the permanence of transcriptionally active mRNA. On this subject, it has been recently reported that the activity of the cellular enzyme TENT5A poly(A) polymerase stabilizes the vaccine mRNA through the addition of up to 200 nucleotides to the mRNA 3′ tail [17]. Interestingly, such an activity was effective mostly in monocyte–macrophages, thus representing a possible mechanism on the basis of the prolonged presence of Spike-expressing monocytes detected in PCVS patients.

With these premises, a widespread biodistribution of the vaccine Spike at variable concentrations in different tissue districts is fairly conceivable.

## 4. Spike-Induced HIV-1 Reactivation: “In Vitro” Studies

The biodistribution of the HIV-1 reservoir in PLWH vaccines can at least in part overlap that of the Spike expressed by COVID-19 mRNA vaccines. Hence, the analysis of the effects of Spike on latent HIV-1 should be considered of outstanding relevance. These have been investigated through “in vitro” studies exploiting well-characterized models of HIV-1 latency in both monocyte–macrophage and lymphocyte human cell lines.

Human monocyte-derived macrophages do not replicate SARS-CoV-2 [18]. However, they react to the interaction with the virus through the release of a large number of soluble factors/cytokines. This phenomenon is restricted to macrophages expressing ACE-2, i.e., the cell membrane receptor binding the SARS-CoV-2 Spike. A couple of papers considered the effects of Spike on U1 cells, i.e., a monocytic–macrophagic cell line where a replication-competent HIV-1 genome remains silent unless the cells are treated with activating stimuli. A first study reported the HIV-1 reactivation in U1 cells after SARS-CoV-2 challenge in a percentage of cells similar to that of cells expressing ACE-2, in the absence of any evidence of SARS-CoV-2 productive infection [19]. This result led the authors to hypothesize that the HIV-1 latency can be broken in macrophages by the cell signaling cascade induced by Spike. This yet largely indirect evidence was, however, corroborated by more recent studies published by Wang and coll. [20], who observed a significant increase in the copy number of HIV-1 RNA in the supernatants of U1 cells treated with soluble Spike compared to control conditions.

In another study, it was reported that the HIV-1 latently infected J-Lat 10.6 lymphocytic cells intracellularly expressing Spike through lentiviral-mediated transduction significantly increased the HIV-1 expression over the background levels [21]. In addition, in this system, soluble Spike synergized with the action of mitogens on latent HIV-1, leading to a further increase in the HIV-1 expression.

Even if some aspects of these studies need further insights, the results are in line with the idea that both extracellular and intracellularly expressed SARS-CoV-2 Spike can force the mechanisms underlying the block of proviral expression in cells latently infected by HIV-1.

## 5. HIV-1 Reactivation Induced by COVID-19 mRNA Vaccines and Spike-Expressing Vectors: “Ex Vivo” Studies

The significance of the results obtained in “in vitro” models has been confirmed and extended by investigations carried out by treating peripheral blood mononuclear cells (PBMCs) from ART-treated PLWH with COVID-19 mRNA vaccines. Stevenson and coll. [22] demonstrated a striking increase in the number of HIV-1 RNA copies released in supernatants of PBMCs from ART-suppressed patients after treatment in culture with either BNT162b2 or mRNA-1273 COVID-19 mRNA vaccines. HIV-1 reactivation occurred in the absence of cell activation and was confirmed in experiments conducted with purified CD4+ T lymphocytes treated with the BNT162b2 vaccine.

Xu and coll. achieved similar results by transducing PBMCs from ART-treated PLWH with a Spike-expressing adeno-associated vector [21]. In this “ex vivo” system, the reactivation of latent HIV-1 was assessed by measuring the transcriptional levels of both LTR and Gag sequences, as well as titrating the viral particles released in the supernatants by challenging an HIV-1 indicator cell line. As expected, the HIV-1 reactivation observed in PBMC cultures was associated with an overall decrease in the CD4+ T lymphocyte sub-population.

The results from these studies consistently demonstrated that both COVID-19 mRNA vaccines as a whole and the Spike protein alone can break the latency of HIV-1 integrated in the DNA of the PBMCs from ART-treated PLWH, leading to the release of infectious viral particles.

## 6. Molecular Mechanisms of HIV-1 Reactivation Induced by COVID-19 mRNA Vaccines and Spike-Expressing Vectors

The effects of the interaction of the COVID-19 mRNA vaccine and the product thereof with HIV-1 latently infected cells in terms of the activation of cell signaling have been a matter of detailed investigations. Bulk mRNA sequencing served to analyze the molecular mechanism underlying the HIV-1 activation observed after treatment with either BNT162b2 or mRNA-1273 COVID-19 vaccines of CD4+ T lymphocytes isolated from ART-treated PLWH [22]. Compared to untreated samples, the BTN162b2 vaccine differentially induced 71 genes, whereas the treatment with the mRNA 1273 vaccine led to the differential activation of 193 genes. It was found that HIV-1 activation was associated with an increase in mRNA-expressing products involved in RIG-I-like receptor-, IFN-, TNF-, and TLR-related cell signaling pathways, in the absence of overt cell activation. Notably, the activation of RIG-I-like receptors is typically induced by viral RNA through a mechanism of innate immunity [23]. Considering the very low levels of ACE-2 receptors expressed by resting/quiescent lymphocytes [24,25], the observed induction was suggestive of the intracellular action of Spike and/or of the vaccine mRNA itself. The latter hypothesis was supported by the data obtained in tumor studies demonstrating the Spike-independent induction of inflammatory factors from immune cells exposed to COVID-19 mRNA vaccines [26].

On the other hand, in J-Lat 10.6 cells, the Spike protein expressed intracellularly reactivated latent HIV-1 through the switch of the mTOR-pS6 pathway, i.e., a driver of several cell metabolism and proliferation processes, as well as of immune responses mediated through the release of inflammatory factors. In particular, it was shown that Spike binds mTOR, thereby promoting its phosphorylation. This event leads to the activation of the downstream pS6 signaling pathway, ultimately driving the induction of the HIV-1 LTR-dependent transcriptional activity [21].

Finally, the results from mechanistic studies carried out on HIV-1 latently infected U1 macrophagic cells [18,19] led to consistent conclusions, although the data were obtained by challenging cells with intact SARS-CoV-2 viral particles.

**Figure 1:**
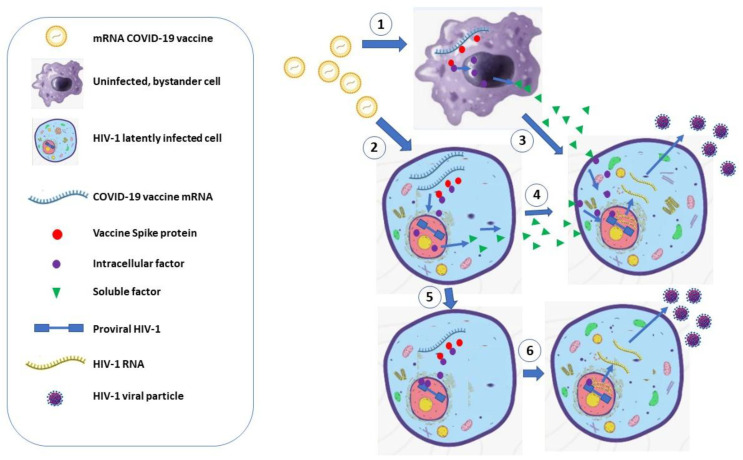
Possible mechanisms of HIV-1 reactivation in latently infected cells by COVID-19 vaccine mRNA. (1). After injection, lipid nanoparticle-complexed mRNA can enter either uninfected (1) or HIV-1 latently infected (2) cells. The combined effects of the mRNA molecules and the Spike protein trigger intracellular signaling, ultimately leading to the release of soluble factors (3, 4), which, in turn, can activate the HIV-1 transcription in latently infected cells through autocrine/paracrine ways. Vaccine mRNA and Spike can also act intracellularly by switching cell signaling. This leads to the induction of LTR-binding transcription factors (5), thereby promoting HIV-1 mRNA transcription and virus production (6).

Together, the results from these studies consistently support the idea that COVID-19 mRNA vaccine-dependent HIV-1 reactivation can be mediated by the induction of inflammatory cytokines driven by the cellular response to both mRNA molecules and Spike protein, as well as by a Spike-induced intracellular activation of HIV-1 LTR (Figure 1).

## 7. Effects of COVID-19 Vaccines in PLWH

A previous case report described the increase in plasma viral load from <50 to 1790 HIV-1 RNA copies in a 65-year-old, ART-suppressed patient after mRNA COVID-19 vaccination [27]. Afterwards, a similar effect was observed in an HIV-1 elite controller not treated with ART [28]. Stevenson and coll. [22] provided evidence that the injection of COVID-19 mRNA vaccines in ART-treated patients induced the activation of Nef- and, to a lesser extent, Rev-specific CD4+ T lymphocytes, a phenomenon reminiscent of the effects observed upon treatment with HIV-1 latency-reversing agents (LRAs). This immune activation occurred in the presence of a slightly reduced cell-associated HIV-1 RNA, most likely a consequence of the cytotoxic action of the HIV-1-specific CD4+ T lymphocytes, and in the apparent absence of changes in the HIV-1 reservoir size when measured through the intact proviral DNA assay (IPDA). However, the vast majority of the proviruses detected with this assay does not reactivate to produce infectious virus even after maximal in vitro stimulation, and some may be limited by chromosomal context from ever reactivating. Based on this assumption, the authors re-evaluated the size of the DNA reservoir with a more specific test, i.e., the Tat/Rev induced limiting dilution assay (TILDA), which quantifies the number of cells able to express Tat/Rev transcripts upon PMA/ionomycin stimulation. This assay allowed researchers to identify two out of four patients where the COVID-19 vaccination was associated with a significant increase in the HIV-1 reservoir size (Table 1).

An investigation on 68 PLWH aged 55 years and older showed that the COVID-19 vaccination was associated with a significant rise in the size of the intact HIV-1 reservoir, as measured by IPDA, in three individuals who did not completely suppress viremia with ART [29]. Conversely, another study performed on 62 vaccinated, ART-treated patients from British Columbia failed to detect any COVID-19 vaccine-related increase in both HIV-1 plasma viremia and reservoir size, as evaluated by IPDA [30] (Table 1).

Finding a unitary interpretation of the results from these three key clinical studies is not obvious, also considering that both times of sampling and endpoints differ among the studies, and some results have been obtained with a limited number of samples. Overall, in the majority of cases, minimal or no effects have been detected in PLWH after COVID-19 vaccination.

To at least in part reconcile the mechanistic findings concerning the effects of COVID-19 vaccines on HIV-1 reactivation with the clinical observations, a number of issues should be considered. First, the ART-induced antiviral pressure can hinder potential increases in plasma HIV-1 RNA. Second, the sampling time is a key factor, since delayed sampling would miss transient effects. Third, the HIV-1 reservoir size has been evaluated mostly with IPDA, i.e., an assay which does not discriminate the actually reactivable HIV-1 proviruses. Fourth, it is conceivable that the size of the HIV-1 reservoir would be the net result of the “de novo” infections together with the killing of cells expressing reactivated HIV-1 by pre-existing HIV-1-specific CD4+ cytotoxic T lymphocytes. Finally, the HIV-1 reservoir is evaluated in blood but not in tissues where ART is less effective, and HIV-1 can spread more easily through cell-to-cell infection.

In conclusion, it seems plausible that current assays devoted to measuring the HIV-1 viremia and the size of the HIV-1 reservoir in ART-treated patients may in some instances overlook the actual virologic consequences of the HIV-1 reactivation following the injection of COVID-19 mRNA vaccines.

## 8. Conclusions

A notable bulk of investigations has been devoted to evaluating the effects of COVID-19 vaccines in PLWH in terms of the induction of Spike-specific immunogenicity. Consistent results showed that the intensity of the anti-Spike immunity response was directly related to the CD4+ T lymphocyte counts [31]. In view of the decreasing impact on health of the infection with currently circulating SARS-CoV-2 variants, another question is gaining relevance for an appropriate cost/benefit analysis of COVID-19 vaccination in PLWH, i.e., what could be the effect of the COVID-19 vaccine on latent HIV-1 infection? Taken together, the results from both “in vitro” and “ex vivo” studies suggest that mRNA COVID-19 vaccines can reactivate HIV-1 through the induction of intracellular, inflammatory-like signaling induced by both the mRNA molecule itself and the Spike protein, as well as through an autocrine/paracrine loop mediated by the release of soluble factors. Additional investigations are anticipated to confirm or refute these conclusions.

Moreover, based on the already available results, relevant issues still await clarification: (i) Both the vaccine mRNA and Spike protein can persist in both plasma and lymph nodes for several days after injection. Furthermore, the Spike protein, both in its full-length and S1 truncated forms, can cross the blood–brain barrier [32,33], thereby entering the CNS. Lymph nodes and the CNS are sites where ART is often less effective [34]. How efficiently can both free and cell-associated Spike induce HIV-1 reactivation in these districts? (ii) The antiviral pressure exerted by ART can limit the systemic viral spread even in the presence of HIV-1 reactivation. Is it possible that after COVID-19 vaccination, the virus diffuses through the virologic synapsis-driven cell-to-cell mechanism, which is a quite efficient way of virus spread particularly under ART [35]? (iii) To what extent can HIV-1-specific cytotoxic CD4+ T lymphocytes, which persist under ART [36] and can be reactivated after COVID-19 vaccination, limit the increase in the HIV-1 reservoir by attacking the newly infected cells? (iv) It has been reported that, in the presence of ART, drug-resistant viruses have a replicative advantage over wild-type ones when spreading through cell-to-cell contact [37]. Can new viral quasispecies emerge after vaccine-induced virus reactivation, considering the intrinsic high mutation propensity of HIV-1?

These still unresolved issues need urgent investigations since, as witnessed by some clinical evidences, HIV-1 reactivation can occur in PLWH with persistent viremia and low CD4+ T lymphocyte counts despite ART, which represents the most “fragile” part of the PLWH population. These patients have the highest priority for COVID-19 vaccination; however, they represent the most vulnerable population in terms of the possible emergence of drug-resistant HIV-1 quasispecies. While the hypotheses discussed here raise potential mechanistic concerns regarding Spike-based vaccines, these considerations await responses and do not necessarily imply that current SARS-CoV-2 vaccination strategies are unsafe for people living with HIV.

In any case, in view of the potentially deleterious effects of Spike on PLWH, limiting/avoiding contact with SARS-CoV-2 would be highly desirable as well. Unfortunately, current COVID-19 vaccines protect quite scarcely from the infection and, following repeated vaccinations, they can generate an overload of circulatory Spike that can persist over time. For these reasons, the ideal COVID-19 vaccine for PLWH would rely on protein, non-Spike COVID-19 immunogens. The recently described vaccine candidate based on the SARS-CoV-2 N protein, and eliciting a cell-based immunity with the generation of N-specific tissue-resident CD4+ T lymphocytes in the airways [38] would be a good option to protect PLWH.

## Figures and Tables

**Table 1:** Summary of the outcomes from clinical studies on COVID-19 vaccinated PLWH.

Reference	Number of Patients	ART Suppression Status	Time of Sampling	Plasma HIV-1 RNA	Cell-Associated RNA	HIV-1 Reservoir Size
Stevenson et al. [22]	13	HIV-1 suppressed for at least one year before vaccination	2 weeks after both first and second vaccinations		1.5- to 1.6-fold decrease compared to baseline values	IPDA: no significant variations throughout; TILDA: Increase after each injection in 2/4 patients
Matveev et al. [29]	68, >55-years aged	63 HIV-1 suppressed patients; 5 with low-level viremia (LLV), >40 copies/mL	24 and 48 weeks after first vaccination			IPDA: no variations except that increase from 35.5 to 175% in 3 LLV at the 48 weeks timepoint
Duncan et al. [30]	62	HIV-1 suppressed (pVL < 20 copies/mL)	4 weeks after both first and second vaccination	No significant/persistent increase. pVL < 200 copies/mL throughout		IPDA: no variations

## Data Availability

No new data were created.

## Abbreviations

The following abbreviations are used in this manuscript:

PLWH	People living with HIV
ART	Antiretroviral therapy
CNS	Central nervous system
PCVS	Post-COVID-19 vaccine syndrome
ACE-2	Angiotensin-converting enzyme-2
PBMCs	Peripheral blood mononuclear cells
LTR	Long terminal repeat
